# Case report: a robotic-vaginal approach for total vaginectomy and hysterectomy with pelvic sentinel lymph node dissection in primary vaginal melanoma: a 10-step technique and literature review

**DOI:** 10.3389/fsurg.2023.1189196

**Published:** 2023-05-26

**Authors:** Philippe Van Trappen, Ines Lebbe, Eveline De Cuypere, Nele Claes

**Affiliations:** ^1^ Department of Gynecology and Gynecological Oncology, AZ Sint-Jan Hospital Bruges, Bruges, Belgium; ^2^ Department of Medical Oncology, AZ Sint-Jan Hospital Bruges, Bruges, Belgium

**Keywords:** hysterectomy, melanoma, robotic, sentinel, vaginal, vaginal melanoma, vaginectomy

## Abstract

**Introduction:**

Primary vaginal melanoma is extremely rare, has a poor prognosis, and occurs mostly in elderly women. The diagnosis is based on histology and immunohistochemistry of a biopsy. Given the rarity of vaginal melanoma, no standardized treatment guidelines are established; however, surgery is the primary treatment modality in the absence of metastatic disease. Most reports in the literature are retrospective single cases, case series, and population-based studies. The open surgical approach is the main modality reported. Here, we report for the first time a 10-step combined robotic-vaginal technique, with *en bloc* resection of the uterus and total vagina, for treating clinically early-stage primary vaginal melanoma. In addition, the patient in our case underwent a robotic pelvic bilateral sentinel lymph node dissection. The literature on the surgical approach for vaginal melanoma is reviewed.

**Case presentation:**

A 73-year-old woman was referred to our tertiary cancer center and was clinically staged according to the 2009 International Federation of Gynaecology and Obstetrics (FIGO) staging for vaginal cancer as FIGO-stage I (cT1bN0M0) and according to the American Joint Committee on Cancer (AJCC) for (cutaneous) Melanoma Staging as clinical stage IB. Preoperative imaging with magnetic resonance imaging, FDG-positron emission tomography-computed tomography, and ultrasound of the groins did not reveal any adenopathy nor metastases. The patient was planned for a combined vaginal and robotic *en bloc* total vaginectomy and hysterectomy, as well as a pelvic bilateral sentinel lymph node dissection.

**Results:**

The surgical procedure was performed in 10 steps described in this case report. The pathology revealed free surgical margins and negative test results for all sentinel lymph nodes. The postoperative recovery process was uneventful, and the patient was discharged on day 5.

**Conclusion:**

The main surgical approach reported for primary early-stage vaginal melanoma is open surgery. A minimally invasive surgical approach, described here as a combined vaginal-robotic *en bloc* total vaginectomy and hysterectomy, for the surgical treatment of early-stage vaginal melanoma enables precise dissection, low surgical morbidity, and fast recovery for the patient.

## Introduction

Malignant melanoma is a highly aggressive tumor that originates from melanocytes, and it arises most commonly from the skin and rarely from the mucosal membranes. Approximately 1.3%–1.4% of all melanomas are of mucosal origin, and 18% arise from the vulvovaginal area (1–4). Vaginal melanoma is a rare type of mucosal melanoma, and its estimated population-based incidence is 0.24–0.26 cases/million/year (5, 6). The most commonly affected women are between 57 and 68 years of age; however, one recent population-based report revealed that the median age at the time of diagnosis is 73 years (5). The common locations of vaginal melanoma are the anterior vaginal wall and the lower third of the vagina. The prognosis of vaginal melanoma is poor, and the major prognostic factors are tumor stage, tumor size, depth of invasion, presence of ulceration, lymph node status, and metastasis (7). The 5-year overall survival rates for vaginal melanoma range between 5% and 25%, compared with 24% and 77% for vulvar melanoma (8, 9). The most common symptoms of vaginal melanoma are vaginal bleeding, abnormal discharge, itchiness, dyspareunia, and pain (10). The final diagnosis is based on pathological, immunohistochemical, and genetic analyses of a biopsy, as well as imaging, to determine the extent of disease. Given the rarity of this type of melanoma, no standardized treatment protocols have been defined, and the guidelines for cutaneous melanoma are often extrapolated to manage vaginal melanoma. In the absence of metastatic disease on imaging and if complete resection is feasible, with free surgical margins, primary surgery is the preferred treatment modality as it is associated with increased survival (4, 11–13). Depending on the surgical pathological findings as well as the immunohistochemistry and/or genetic analyses, adjuvant therapy can be considered on an individual basis, with immunotherapy and targeted therapy in the presence of genetic alterations being the preferred treatments of choice (4, 11–13).

Here, we describe for the first time a 10-step surgical procedure of a combined vaginal and robotic *en bloc* total vaginectomy and hysterectomy, with pelvic sentinel lymph node (SLN) dissection, for treating primary vaginal melanoma. The recent literature on the surgical approach for vaginal melanoma is reviewed.

## Case report

A 73-year-old European woman presented with abnormal vaginal discharge. On clinical vaginal examination, a brown pigmented flat lesion of 2 × 2 cm was seen on the posterior vaginal wall just beyond the vaginal introitus. The lateral and anterior walls of the vagina did not show any abnormalities. No inguinal lymphadenopathy was found. Her medical history included only hypertension. She was a non-smoking female, gravida 4—para 4, with four vaginal deliveries. Her surgical history included a bilateral salpingo-oophorectomy and an appendectomy. Her family history showed her mother died of metastatic breast cancer.

A primary excisional biopsy was performed, and pathology reports showed a malignant melanoma with a minimal diameter of 20 mm and a thickness of 8 mm. There was no lympho-vascular or perineural invasion. The margins were positive on the lateral and distal sides. The patient was subsequently referred to our tertiary cancer center for robotic oncological surgery. Preoperative imaging involved magnetic resonance imaging (MRI), ultrasound scan of the groins, and FDG-positron emission tomography/computed tomography (PET-CT) scan. All of these investigations did not reveal any lymphadenopathy nor distant metastases. To exclude primary melanoma from other sides such as ocular melanoma, an ophthalmologic investigation was performed, which did not reveal any pathology. On the basis of these investigations, a primary melanoma from another side was excluded. The patient was clinically staged according to the 2009 International Federation of Gynaecology and Obstetrics (FIGO) staging for vaginal cancer as FIGO-stage I (cT1bN0M0) and according to the American Joint Committee on Cancer (AJCC; eighth edition) for (cutaneous) Melanoma Staging as clinical stage IB (T4bN0M0, with T4: >4 mm thickness and b: with ulceration) (14–16). The patient was planned for a combined vaginal and robotic *en bloc* resection of the total vagina and uterus, in order to achieve a complete resection of the vaginal melanoma, together with a pelvic sentinel lymph node dissection.

The robot da Vinci Xi platform (Intuitive Surgical Inc., Sunnyvale, CA, USA) with near-infrared fluorescence imaging (Firefly® technology) was used. As a fluorescent agent, indocyanine green (ICG: Verdye 25 mg, Diagnostic Green GmbH ©, 85609 Aschheim-Dornach, Germany) was used at a concentration of 1.9 mg/mL H_2_O. A total of 2 mL ICG was used for the patient and 0.5 mL was injected, just beyond the vaginal introitus, in the submucosa of the anterior, posterior, and lateral vaginal walls.

## Surgical technique

**Figure 1:**
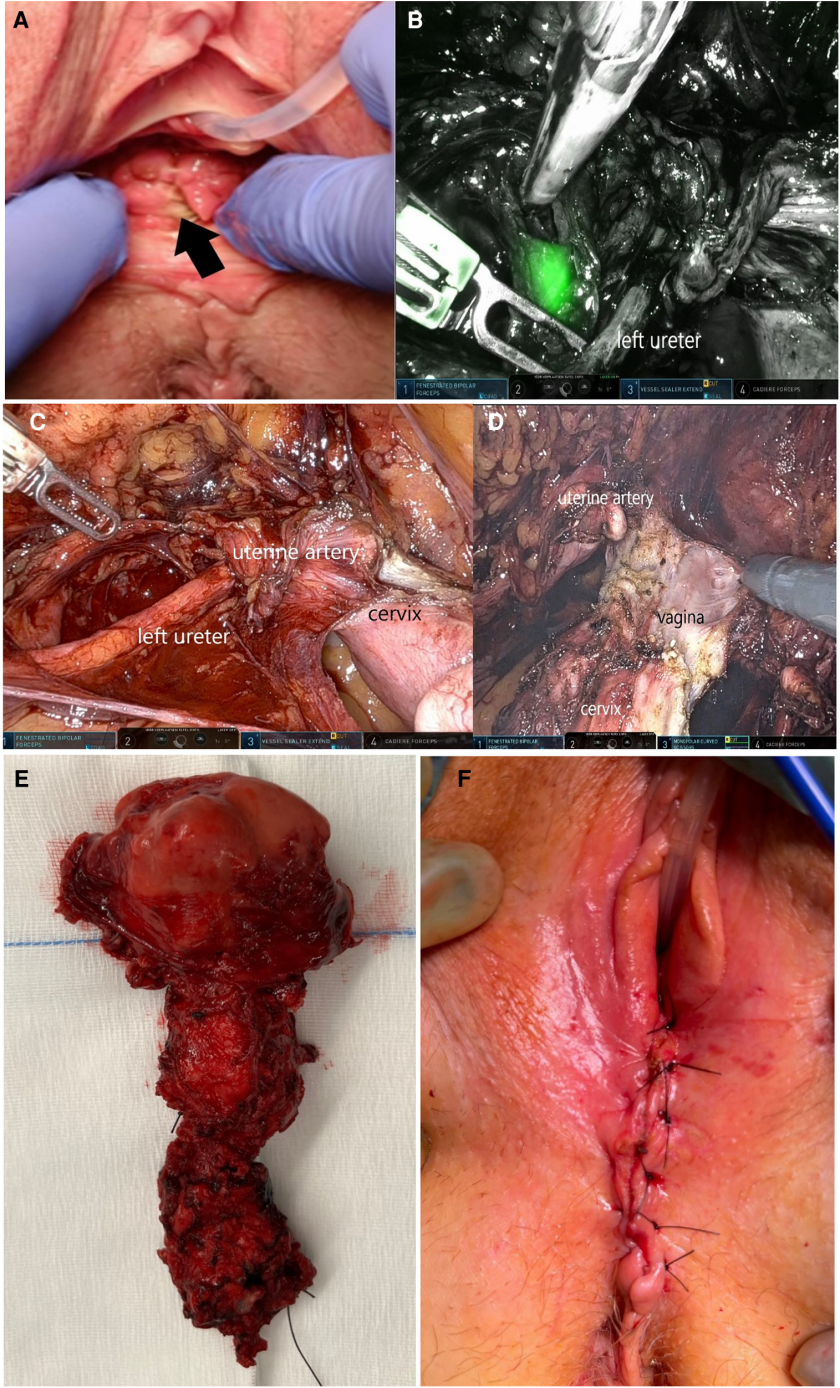
(**A**) Clinical examination shows a pigmentated flat lesion of 2 × 2 cm on the lower posterior wall of the vagina; (**B**) pelvic sentinel lymph node at the left internal iliac vessels, identified with near-infrared (Firefly®) imaging during robotic surgery; (**C**) ureterolysis on the left side, down to the level of the crossing with the uterine artery; (**D**) dissection of the anterior and lateral vagina; (**E**) *en bloc* specimen of the combined vaginal and robotic resection of the uterus and complete vagina; and (**F**) closure of the vaginal introitus during surgery.

A robotic pelvic bilateral sentinel lymph node dissection was performed prior to the *en bloc* resection of the uterus and total vagina (Figure 1).

The 10 surgical steps of the combined vaginal and robotic *en bloc* total vaginectomy and hysterectomy were as follows, with the patient placed in the lithotomy position (Figure 1):

1. Vaginal approach: circular incision, with diathermy, of the vaginal mucosa just inside the vaginal introitus.
2. Subsequent dissection of the distal 3 cm of vagina, with releasing the vagina anteriorly from the urethra, posteriorly from the rectum, and laterally on both sides from the bulbocavernosus muscle.
3. Robotic approach: sealing/cutting of the round ligaments with an opening of the retroperitoneal space at the pelvic side walls.
4. Ureterolysis: traction medially on the peritoneum of the broad ligament with subsequent ureterolysis, while keeping the mesoureter intact, down to the level of the crossing with the uterine arteries.
5. Sealing of the uterine arteries at its origin, close to the internal iliac artery, and lateral from the ureters.
6. Traction of the bladder peritoneum toward the anterior abdominal wall (with robotic Cadière forceps) and sealing/cutting of the uterine arteries on the uterus, both ascending and descending branch down to the vaginal edges.
7. Preparation for the robotic vaginal dissection: with fenestrated grasper, via the assistant trocar, traction of the uterus cranially, and with the robotic Cadière foceps traction of the bladder peritoneum toward the anterior abdominal wall.
8. Anterior/lateral robotic vaginal dissection: dissection and dividing of the vagina from the bladder anteriorly and paravaginal tissues laterally, with robotic bipolar forceps and unipolar scissor, down to the level of the vaginal dissection.
9. Posterior robotic vaginal dissection: with the robotic Cadière forceps elevation of the uterus against the pubis; opening of the posterior peritoneum with unipolar scissor and sealing/cutting of the sacro-uterine ligaments; dissecting the rectovaginal septum with releasing the total vagina from the rectum down to the level of the initial vaginal dissection; removal of the total vagina and uterus vaginally.
10. Closure of the vaginal introitus: approximation of the bulbocavernosus muscle on both sides toward the midline with Vicryl 1 sutures, below the level of the urethral orifice. Closure of the skin above the bulbocavernosus muscle with Ethilon 2/0 Donati stitches.

The methylene blue test showed a small bladder lesion at the top of the bladder, which was closed with two single sutures Monocryl 3/0. The estimated blood loss was approximately 300 cc. The rectal serosa was intact. The total combined vaginal and robotic operation time was 195 min. Postoperative recovery was uneventful. Hemoglobin levels did not drop below 9.8 g/dL. C-reactive protein levels remained low postoperatively (maximum of 13 mg/L). The patient was discharged on day 5. The bladder catheter was removed during an outpatient visit on day 10. She had no complaints. The pathology report showed a residual malignant melanoma of 6.23 mm in width with an invasion thickness of 0.8 mm; the combined dimensions of the residual melanoma and the initial excision biopsy (20 x 8mm, with ulceration) provide a final TNM stage as pT4bN0 (AJCC Stage IB) or FIGO stage I. There was no ulceration in the residual melanoma, perineural and lympho-vascular invasion were absent, and there was no invasion in the cervix. The surgical margins were free. All bilateral sentinel lymph nodes showed negative results. Immunohistochemical staining showed that the tumor cells were positive for Sox10, PRAME, and MIB1. P53 showed a wild-type expression. PHH3 did not show a clear mitosis. ATRX showed a weak but preserved expression in tumor cells. No BRAF mutation was found; however, a mutation was found in the KIT gene (exon 11). The suggested multidisciplinary advice was adjuvant treatment with immunotherapy and an intravenous administration of Pembrolizumab (PD-1 receptor blocker) for 1 year. During follow-up, there was no evidence of recurrent disease clinically or on imaging 4 months after surgery.

## Discussion

Primary vaginal melanoma is extremely rare and no standardized treatment guidelines are established; however, surgery is the primary treatment modality in the absence of metastatic disease. In the patient in our case, imaging with MRI, PET-CT, and ultrasound of the groins did not reveal any metastatic disease nor lymphadenopathy, and the primary treatment was a combined vaginal and robotic *en bloc* total vaginectomy and hysterectomy, including pelvic SLN dissection, with low morbidity and fast recovery.

Primary vaginal melanoma is an aggressive type of tumor with poor prognosis, which occurs mainly in older women, and due to the anatomical location, the diagnosis is often made at a late stage of the disease, with 50% of patients having nodal involvement at diagnosis (17, 18). In the early stages, surgery can control the tumor as part of initial therapy.

A pathological examination of a biopsy is crucial in the final diagnosis, and histologically, these tumors may show epithelioid and spindle neoplastic cells in pure or mixed populations. Immunohistochemistry is useful in the differential diagnosis of vaginal melanoma when specific markers such as S-100, HMG-45, SOX10, and MelanA are employed (17, 19). While BRAF and NRAS mutations are common pathogenic mutations in cutaneous melanoma, mucosal melanomas usually lack BRAF and NRAS mutations, and instead have KIT alterations, as described in our case. The most common mutation spots in vaginal melanoma are found in the KIT, TP53, ATRX, and NF1 genes.

The open surgical approach for primary vaginal melanoma has been the main treatment modality, as shown in previous reports (Table 1). The number of cases included in retrospective studies and case series ranged between 1 and 230. The median overall survival was 16–39.5 months, and the 5-year overall survival rates varied between 15.4% and 32.3%. Surgery is the primary treatment modality in the absence of metastatic disease. However, it has been found that a radical surgical approach (radical vaginectomy/hysterectomy) has no significant impact on overall survival, but is associated with increased morbidity (11, 29). The main aim of primary surgery is to achieve complete resection with free margins on histology, as in our case (7). Metastatic disease, after primary surgical treatment, occurs irrespective of the radicality of surgery. In clinical early-stage vaginal melanoma with no evidence of lymphadenopathy on radiology, routine groin and pelvic lymph node (LN) dissection is not recommended, because in such patients, the incidence of LN metastasis is low. Hence, in our patient, no suspicious lymph nodes were seen on preoperative imaging and only the pelvic bilateral sentinel lymph nodes were removed (which were negative on histology), which bore similarities to a few previous reports (5, 28).

**Table 1:** Surgical management and adjuvant treatment for primary vaginal melanoma.

Author	Year	Surgery	Number	Adjuvant treatment	Outcome
		Open/laparoscopic			
Geisler et al. (20)	1995	Open	4	None	1 patient died
Cobellis et al. (18)	2000	Open	15		Median OS: 19 months
Gökasian et al. (21)	2005	Open	1	IM	died
Betschart et al. (22)	2007	Open	1	RT/CT	Recurrence after 6 months
Ghosh et al. (23)	2007	Open	1	RT/CT	Died after 6 months
				IM	
Fukui et al. (24)	2008	Open	1	IM	No recurrence
Biswas et al. (25)	2009	Open	1	RT/CT	Unknown
Greggi et al. (26)	2010	Open	2	IF	Both died
Vaysse et al. (27)	2013	Open	42	RT/CT	Median OS: 28.4 months
Kirschner et al. (28)	2013	Open	87	RT	5-year OS: 15.4%
Huang et al. (29)	2013	Open	22	CT/IM	5-year OS: 32.3%
Xia et al. (6)	2014	Open	41	RT/CT	Median OS 39.5%
Seifried et al. (30)	2015	Open	11	RT/CT	5-year Survival: 48%
Wohlmuth et al. (31)	2020	Open	230	IM	Median OS: 16 months
Khayyat et al. (13)	2022	Open	8	RT/CT	2 died; 1 recurrence
				IM	
Yazdanfard et al. (5)	2022	Open/laparoscopic	38	RT/CT	5-year OS: 19.4%
				IM	
Tian et al. (11)	2022	Open	62	CT/IM	Median OS: 25.9%
				IF/TT c-KIT	

OS, overall survival; IM, immunotherapy; RT, radiotherapy; CT, chemotherapy; IF, interferon therapy; TT, targeted therapy.

Minimally invasive total vaginectomy with hysterectomy has been reported in a few case series of female-to-male genital reassignment surgery, which is mainly related to the laparoscopic approach, with only one report on robotic surgery (32, 33). However, robotic surgery for malignant melanoma has been described only for locally advanced vulvovaginal melanoma, with the surgical approach being an anterior pelvic exenteration (34). In our case, we describe a combined vaginal and robotic approach with total vaginectomy and hysterectomy, with sentinel lymph node dissection, for treating clinically early-stage vaginal melanoma. In addition to the advantages of laparoscopic surgery compared with open surgery, in terms of perioperative complications, using a robotic platform provides the surgeon additional benefits such as 3D vision, precise dissection with enlarged vision, and freedom to use different surgical instruments.

Postoperative adjuvant therapy has a positive influence on overall survival in vaginal melanoma, as demonstrated recently by the report of Tian et al. (11). The preferred adjuvant treatment in vaginal melanoma is immunotherapy, such as anti-PD1 monotherapy with pembrolizumab or nivolumab, or targeted therapy for the mutations present (35). These therapies are also the preferred first-line treatments for metastatic or unresectable vaginal melanoma. Chemotherapy has limited impact on overall survival and is therefore not the preferred first-line treatment. In our case, the patient received immunotherapy as adjuvant therapy with pembrolizumab.

## Conclusion

Here, we report for the first time a 10-step combined vaginal and robotic technique, with *en bloc* resection of the uterus and complete vagina, for treating clinically early-stage primary vaginal melanoma. In addition, the patient underwent a robotic pelvic bilateral sentinel lymph node dissection. The limitations of this study are linked to the fact that it is only a case report. The strength is that this novel combined robotic-vaginal technique enabled precise dissection with low morbidity and fast recovery for the patient.

## Data Availability

The original contributions presented in the study are included in the article, further inquiries can be directed to the corresponding author.
